# Draft Genome Sequence of a Selenite- and Tellurite-Reducing Marine Bacterium, *Lysinibacillus* sp. Strain ZYM-1

**DOI:** 10.1128/genomeA.01552-15

**Published:** 2016-01-14

**Authors:** Yonghe Zhao, Yuxuan Dong, Yiwen Zhang, Lin Che, Haixia Pan, Hao Zhou

**Affiliations:** Key Laboratory of Industrial Ecology and Environmental Engineering (Ministry of Education), School of Food and Environment, Dalian University of Technology, Panjin, China

## Abstract

*Lysinibacillus* sp. ZYM-1, a Gram-positive strain isolated from marine sediments, reduces selenite and tellurite efficiently. Meanwhile, it also exhibits high resistance to Zn2+ and Mn2+. Here, we report the draft genome sequence of strain ZYM-1, which contains genes related to selenite and tellurite reduction and also metal resistance.

## GENOME ANNOUNCEMENT

*Lysinibacillus* is a Gram-positive, rod-shaped, and round-spore-forming bacterial genus in the family *Bacillaceae* (1). More recently, several reports have indicated that *Lysinibacillus* spp. are potential candidates for heavy metal bioremediation. *L. fusiformis* ZC1 can reduce 1 mM hexavalent chromium within 12 h, and 25 strains of *L. sphaericus* can grow in arsenate, hexavalent chromium, and/or lead (2, 3). Strain ZYM-1 was isolated from marine sediments using 1 mM selenite as selection pressure. This strain has been deposited in the China General Microbiological Culture Collection Center (accession number: CGMCC 1.15346). The 16S rRNA sequences of strain ZYM-1 (GenBank accession number: KT263530) revealed that it belongs to the genus *Lysinibacillus*. Besides the widely reported heavy metal resistance of *Lysinibacillus* spp., strain ZYM-1 also exhibits high selenate (MIC 10 mM), selenite (MIC 100 mM), and tellurite (MIC 2 mM) resistance. Red selenium and black tellurium nanoparticles were formed as reduction products of selenite and tellurite, which have potential application in heavy metal adsorption, photocatalysis, and energy storage (4–6). As no selenite/tellurite reduction capacity has been reported among the *Lysinibacillus* spp., and most strains of *Lysinibacillus* have been isolated from soils, the genome sequence of strain ZYM-1 may provide fundamental information of selenite/tellurite reduction genes in this species.

The genome of strain ZYM-1 was sequenced using Illumina HiSeq-2500 by PE125 strategy. The obtained reads were assembled into 84 large contigs using SOAPdenovo software (http://soap.genomics.org.cn/soapdenovo.html). Then, gene prediction was performed using the GeneMarkS server (http://opal.biology.gatech.edu). The genome sequence of ZYM-1 is 4,862,873 bp in length with G+C content of 37.86%. There are 5,006 predicted coding sequences, accounting for 85.01% of the total sequences.

A rich set of 247 annotated genes are related to inorganic ion transport and metabolism according to the COG function classification (7). As mentioned previously, strain ZYM-1 could tolerate selenite up to 10 mM, but failed to reduce this oxyanion. This is consistent with the absence of any reported genes encoding selenate reductases (*ser*ABC, *srd*BCA, *ynf*E, and *ygf*K) in the genome. In addition, the genes encoding NirS-type nitrite reductases are also not found (8). However, five thioredoxin reductases encoding genes are identified, which may have mediated the selenite reduction in strain ZYM-1 (9, 10). Also, two genes (*ded*A and *cys*A) encoding the possible uptake proteins selenite and selenate were found. On the other hand, we also found several genes involved in tellurite resistance and reduction, including *ter*C, *ter*D, *yce*H, and *yce*F (11). In addition, there are several genes involved in heavy metal resistance, such as ABC-type Mn2+/Zn2+ transport systems (*ytg*A, *ytg*B, *ytg*C, and *ytg*D) and the Zn2+ responsive regulator *zur*, which may relate to the Mn2+/Zn2+ resistance of strain ZYM-1. The genome sequence information indicates that strain ZYM-1 can provide a platform for selenite/tellurite detoxification and production of selenium/tellurium nanoparticles.

### Nucleotide sequence accession numbers.

This whole-genome shotgun project has been deposited at DDBJ/EMBL/GenBank under accession number LKPY00000000. The version described in this paper is the first version, LKPY01000000.

## References

[1] Nam Y-D, Seo M-J, Lim S-I, Lee S-Y 2012 Genome sequence of *Lysinibacillus boronitolerans* F1182, isolated from a traditional Korean fermented soybean product. J Bacteriol 194:5988. doi:10.1128/JB.01485-12. 23045499 PMC3486081

[2] He M, Li X, Liu H, Miller SJ, Wang G, Rensing C 2011 Characterization and genomic analysis of a highly chromate resistant and reducing bacterial strain *Lysinibacillus fusiformis* ZC1. J Hazard Mater 185:682–688. doi:10.1016/j.jhazmat.2010.09.072. 20952126

[3] Lozano LC, Dussán J 2013 Metal tolerance and larvicidal activity of *Lysinibacillus sphaericus*. World J Microbiol Biotechnol 29:1383–1389. doi:10.1007/s11274-013-1301-9. 23504213

[4] Jain R, Jordan N, Schild D, van Hullebusch ED, Weiss S, Franzen C, Farges F, Hübner R, Lens PNL 2015 Adsorption of zinc by biogenic elemental selenium nanoparticles. Chem Eng J 260:855–863. doi:10.1016/j.cej.2014.09.057.

[5] Nath S, Ghosh SK, Panigahi S, Thundat T, Pal T 2004 Synthesis of selenium nanoparticle and its photocatalytic application for decolorization of methylene blue under UV irradiation. Langmuir 20:7880–7883. doi:10.1021/la049318l. 15323543

[6] Kim MG, Kim D, Kim T, Park S, Kwon G, Kim MS, Shin TJ, Ahn H, Hur HG 2015 Unusual Li-ion storage through anionic redox processes of bacteria-driven tellurium nanorods. J Mater Chem A 3:16978–16987. doi:10.1039/C5TA04038H.

[7] Tatusov RL, Galperin MY, Natale DA 2000 The COG database: a tool for genome-scale analysis of protein functions and evolution. Nucleic Acids Res 28:33–36. doi:10.1093/nar/28.1.33. 10592175 PMC102395

[8] Bertolini C, van Aerle R, Lampis S, Moore KA, Paszkiewicz K, Butler CS, Vallini G, van der Giezen M 2014 Draft genome sequence of *Stenotrophomonas maltophilia* SeITE02, a gammaproteobacterium isolated from selenite-contaminated mining soil. Genome Announc 2(3):e00331–14. doi:10.1128/genomeA.00331-14. 24812214 PMC4014682

[9] Björnstedt M, Odlander B, Kuprin S, Claesson H, Holmgren A 1996 Selenite incubated with NADPH and mammalian thioredoxin reductase yields selenide, which inhibits lipoxygenase and changes the electron spin resonance spectrum of the active site iron. Biochemistry 35:8511–8516. doi:10.1021/bi9528762. 8679612

[10] Li X, Kot W, Wang D, Zheng S, Wang G, Hansen LH, Rensing C 2015 Draft genome sequence of Se(IV)-reducing bacterium *Pseudomonas migulae* ES3-33. Genome Announc 3(3):e00406–15. doi:10.1128/genomeA.00406-15. 25953191 PMC4424307

[11] Taylor DE 1999 Bacterial tellurite resistance. Trends Microbiol 7:111–115. doi:10.1016/S0966-842X(99)01454-7. 10203839

